# Can Computed Tomography Perfusion Predict Treatment Response After Prostate Artery Embolization: A Feasibility Study

**DOI:** 10.3390/diagnostics10050304

**Published:** 2020-05-15

**Authors:** Brian Malling, Martin Andreas Røder, Carsten Lauridsen, Lars Lönn

**Affiliations:** 1 Department of Diagnostic Radiology, Rigshospitalet, Blegdamsvej 9, 2100 Copenhagen, Denmark; brian.malling.01@regionh.dk (B.M.); carsten.ammitzboel.lauridsen.01@regionh.dk (C.L.); Lars.birger.loenn@regionh.dk (L.L.); 2 Department of Urology, Rigshospitalet, Blegdamsvej 9, 2100 Copenhagen, Denmark; 3 Bachelor’s Degree Programme in Radiogeltraphy, Copenhagen University College, Sigurdsgade 26, 2200 Copenhagen, Denmark

**Keywords:** tomography, X-ray computed, four-dimensional computed tomography, embolization, therapeutic, prostatic diseases, clinical trial, prostatic neoplasms, prostatic hyperplasia

## Abstract

Prostate artery embolization (PAE) is an emerging therapy for benign prostatic hyperplasia (BPH). Optimal patient selection is an important step when introducing new treatments and several characteristics associated with a good clinical outcome has previously been proposed. However, no prognostic tool is yet available for PAE. Computed tomography perfusion is an imaging technique that provides hemodynamic parameters making it possible to estimate the prostatic blood flow (PBF). This study investigated the relationship between PBF and the response to PAE. A post hoc analysis including prostate-specific antigen (PSA) measurements before and 24-h after embolization from two prospective studies on sixteen patients undergoing PAE with BPH or prostate cancer were performed. The primary outcome was the correlation between baseline PBF and the change in PSA as a surrogate measure of treatment response. Prostate volume strongly correlated with treatment response and the response was greater with incremental amounts of injected embolic material. PBF was not associated with elevation in PSA and added no information that could guide patient selection.

## 1. Introduction

Prostate artery embolization (PAE) is a minimally invasive treatment for lower urinary tract symptoms (LUTS) secondary to benign prostatic hyperplasia (BPH) [1,2]. The primary mechanism of action is ischemia, inflammation and necrosis by occluding the prostatic blood supply with small embolic particles, and subsequent shrinkage of the prostate [3]. It has also been speculated that androgen-related apoptosis play a role in the volume reduction of the prostate and the relief of bladder outlet obstruction [3]. The prostatic injury following PAE causes a transient increase in prostatic-specific antigen (PSA) which correlates with the extend of prostatic infarction and magnitude of symptomatic improvement [4,5,6,7]. However, since change in PSA can only be calculated once embolization has been performed, it is not useful for patient selection. Accordingly, there is a need to identify patient characteristics that can predict clinical outcome following treatment.

International guidelines from interventional radiology societies recommend PAE in men with BPH and moderate-severe LUTS including large prostates (>80 mL), acute or chronic urinary retention and hematuria of prostatic origin based on current evidence [8]. BPH is a histological diagnosis characterized by glandular-stromal growth predominately in the transition zone [9]. The transition zone—and the BPH nodules in particular—is well-perfused, which may explain why these areas are more susceptible to embolization compared to the other zones of the prostate [3]. Evidence suggest that men with prostates dominated by adenofibromatous nodules have a greater therapeutic effect after PAE than men without [10].

Hemodynamic parameters such as blood flow, permeability, blood volume and mean transition time can be measured using a 4-dimensional imaging technique (adding time to the 3D volume acquisition) called computed tomography perfusion (CTP) [11]. The technique is used clinically in the evaluation of patients with ischemic stroke to identify tissue at risk and predict the response to reperfusion [12]. Further, CTP is feasible in the setting of prostate imaging and early evidence suggests that the technique can detect prostate cancer (PCa) [13]. Computed tomography angiography is routinely used in the evaluation prior to endovascular procedures and adding a CTP protocol could readily be implemented in clinical practice [14,15]. To the best of our knowledge, the relationship between treatment response and blood flow has not been investigated before and could potentially guide the patient selection process and reduce the risk of clinical failures.

## 2. Materials and Methods

### 2.1. Study Design

This was an explorative study in a subset of patients nested within two prospective single-center trials conducted by the Department of Radiology and Urology at Rigshospitalet, Copenhagen University Hospital between March 2017 and November 2018 [16,17]. The trial was conducted in accordance with the World Medical Association Declaration of Helsinki. The institutional review board approved the trial protocol (ID: H-17000714, 22 March 2017). Informed written and verbal consent was obtained from all participants.

### 2.2. Study Population

Men with urinary retention or LUTS secondary to BPH or PCa not eligible for TURP or curative treatment were enrolled. Exclusion criteria included allergies to contrast medium, renal insufficiency (eGFR < 45), urethral stricture, large bladder diverticula or stones, bladder neck contracture and known sphincter anomalies.

### 2.3. CTP Protocol

The same 320-detector row CT scanner (Aquilion One, Toshiba Medical Systems) was used for all CTP examinations four weeks before embolization. Using a pump injector (Medrad Stellant, Bayer Healthcare), 50 mL non-ionic contrast medium (Omnipaque 350, GE Healthcare) and 50 mL of saline was administered through an 18-gauge catheter placed in the antecubital vein at a flow rate of 6 mL/s. Bolus triggering in the aorta just above the aortic bifurcation and a 13-s delay was applied. The remaining scanning parameters were as follows: 100 kV, 11049 mA, collimation 0.5 cm × 80 rows, gantry rotation time 0.275 s, matrix 512 × 512, 0.5 mm reconstructed slice thickness, and a volume scan image reconstruction with an adaptive iterative dose reduction 3D (AIDR 3D-standard) algorithm. The protocol included 19 acquisitions divided into three continuous sequences: The first sequence consisted of nine volumes with a two-second scan interval, the second sequence of six volumes with a scanning interval of three seconds and the third sequence of four volumes with a five-second scan interval. The total dose-length product was 758.1 mGy∙cm—equivalent to an effective dose of 11.4-mSv (k-factor 0.015) [18].

**Figure 1:**
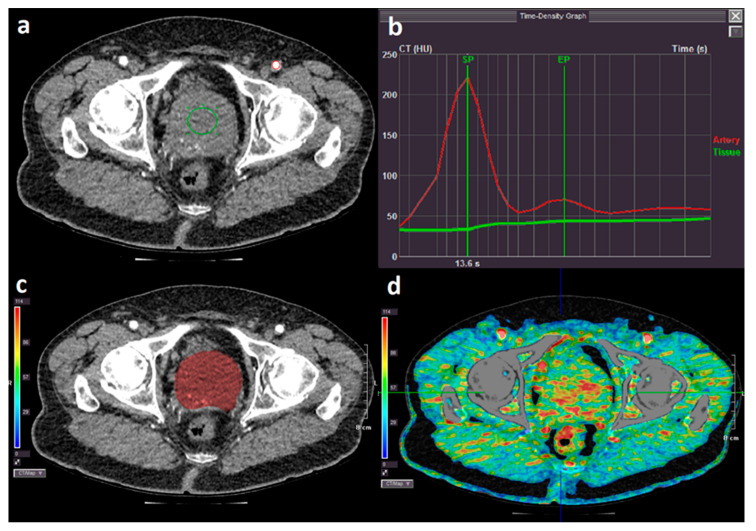
(**a**) 50 mm^2^ and 600 mm^2^ region of interest (ROI) was placed in the left femoral artery (red circle) and centrally in the prostate (green circle) to obtain the arterial and tissue input function, respectively; (**b**) time-density graph showing arterial (red line) and tissue (green line) contrast medium attenuation. The green vertical lines mark the start point (SP) and end point (EP) for perfusion measurements; (**c**) contour of the prostate was outlined manually using the sculpting tool (red area); (**d**) colorimetric map of blood flow.

Postprocessing of the CTP studies was performed on commercially available CTP software (Vitrea, CT body perfusion 4D, Vital Images, Inc.) applying software pre-sets with a processing threshold between 150 and −80 Hounsfield units. Two circular regions of interest (ROI) were manually drawn—one with a size of 50 (±5) mm^2^ in the femoral artery, and one of 600 (±5) mm^2^ placed centrally in the prostate—to obtain the arterial and tissue input function, respectively. The software automatically derived the time attenuation curve (also known as time-density graph) for a maximum slope single input-single compartment model used to reduce scanning time and the risk of motion artefacts [19]. The start phase point was set to the time point of contrast accumulation in the prostatic tissue, and end phase point was set to the maximum attenuation of the prostatic tissue before the second pass of contrast medium. The software generated a colorimetric map showing PBF in the unit mL/min/100 mL. The mean PBF was estimated using the 3D volume of interest sculpting tool (Figure 1a–d).

### 2.4. Embolization Technique

The technique has been described in details elsewhere [20]. In short, embolic particles were injected proximally in the prostate artery and after achieving stasis of the flow, the microcatheter was advanced into the intraprostatic branches. The purpose of initial flow-directed embolization followed by complete occlusion of the microcirculation was to induce a significant infarction. Perioperatively, 5000 units of heparin in bolus were administered followed by 1000–2000 units per hour on the discretion of the operator. Under local anesthesia, a 5F sheath (Fortress, Biotronik) was employed to access the right common femoral artery. The prostate arteries were localized on each hemipelvis and super-selectively catheterized with a 2.0-F microcatheter (Progreat, Terumo Interventional Systems). Before manually injecting 300–500-μm tris-acryl gelatine microspheres (Embosphere, Merit Medical) particles under fluoroscopic guidance, a cone-beam CT (Allura, Phillips) was performed to confirm catheter placement and visualize any aberrant collaterals. Branches not supplying the prostate were protected by coil embolization or by advancing the microcatheter distally to their origin before injection of embolic particle suspension. Every milliliter of particle suspension injected was followed by three milliliters of saline.

### 2.5. Outcomes Measures

A medical interview and CTP measurements including PBF, prostate volume and PSA were performed at baseline and repeated one month after embolization. Change in PSA was calculated using blood samples taken 24 h after embolization and subtracting baseline values. The change in PSA was used as a surrogate marker of treatment response.

The primary outcome was the correlation between baseline PBF and the change in PSA. Secondary outcomes included changes from baseline to 1-month follow-up in PSA, PBF and prostate volume measured on CTP studies. Correlations coefficients between PBF, prostate volume reduction and embolic particles were calculated.

The perioperative details including procedure duration, fluoroscopy time, dose area product (radiation exposure to the patient), amount of iodine contrast medium, and the administered volume of particle suspension were recorded.

### 2.6. Statistical Analysis

The analysis was performed using R statistical software version 3.6.3 for Mac OS X. Medians with minimum and maximum values were reported for continuous variables. The Wilcoxon signed-rank test was used to test the difference between baseline and follow-up measurements. The R package “Hmisc” and “rcorr” function was applied to calculate the Spearman’s rank correlation coefficient (ρ) ranging from −1 to 1, where 0 indicates no correlation, and 1 and -1 a perfect positive and negative correlation, respectively. Cutoff points suggested elsewhere where used for negligible, weak, moderate or strong correlation: 0.00–0.10 was considered negligible, 0.10–0.40 weak 0.40–0.70 moderate, 0.70–0.90 strong and 0.90–1.00 very strong correlation [21]. A *p*-value < 0.05 was considered statistically significant.

## 3. Results

A total of 16 patients with BPH (*n* = 7) or PCa (*n* = 9) with a median age of 72,5 years (range 64. 1–88.7) underwent CTP before and one month after treatment. The indication for PAE was symptomatic LUTS with an IPSS of 21 (range 5–35) in eight patients and catheter dependency in the remaining eight. Gleason score at diagnosis in the nine men with PCa was 6 (*n* = 2), 7 (*n* = 3), 8 (*n* = 2), 9 (*n* = 1) and 10 (*n* = 1). Four patients had metastatic disease. Baseline PBF and PSA was similar between the two groups (*p*-value 0.17 and 0.68, respectively). Baseline characteristics and 1-month follow-up measurements grouped by patients with BPH, PCa and overall are shown in Table 1.

The change in PSA after embolization did not correlate with baseline PBF. No correlation between PBF and prostate volume reduction was observed (overall correlation coefficient 0.18 and *p*-value 0.5). There was a strong and significant correlation between the change in PSA 24 h after embolization and baseline prostate volume in the PCa and overall group. Overall, the change in PSA correlated strongly with the reduction in prostate volume reduction observed 1 month after treatment. Baseline prostate volume—as well as the number of embolic particles administered—had a moderate or strong correlation with the change in PSA. The Spearman’s rank correlations coefficients between 24-h change in PSA and selected variables are shown in Table 2.

PBF was not significantly different one month after treatment compared to baseline in any group. The change in PSA was significant in the overall and BPH group immediate after embolization, but not at 1-month follow-up.

The perioperative data were as follows: procedure duration and fluoroscopy were 153 min (range 114–270) and 51 min (range 23–76), respectively. The amount of embolic particle suspension and contrast medium administered was 4 mL (range 2–14) and 113 (range 80–170), respectively. The dose-area product was 969 (range 454–2344) Gy∙cm^2^.

## 4. Discussion

This explorative study investigated the correlation between baseline prostatic blood flow (PBF) and the immediate change in PSA following embolization. Earlier reports have established the association between incremental increase in PSA, extent of prostatic infarction and the probability of a good clinical outcome [4,5,6]. These findings support using PSA as a surrogate measure of treatment response. We hypothesized that men with a high PBF would be particular susceptible to therapeutic embolization achieving greater prostatic injury and consequently elevated PSA. Successful embolization was achieved in the overall and BPH group reflected by the significant 24-h PSA elevation and prostate volume reduction. However, no correlation was observed between these outcomes and baseline PBF suggesting that the measure does not add any prognostic information in the patient assessment before PAE. Intuitively, a reduction in PBF due to ischemia could be expected following PAE. However, CTP measures blood flow in the prostate (capillary beds) as the volume of blood delivered to a volume of tissue in a given time (mL/min/100 mL) in contrast to the velocity (i.e., mL/s) measured in larger vessels [22]. Since necrotic tissue and cytotoxic edema is gradually absorbed it is only the volume of less affected tissue that is evaluated on a follow-up scan [3]. Therefore, change in PBF is not a relevant measure of prostatic injury and accordingly no reduction in PBF was observed.

Prostate volume was the only baseline variable that had a strong overall correlation with the change in PSA. This has been demonstrated in other studies as well where better clinical outcomes in men with larger prostates compared to men with smaller prostates have been shown [23,24]. Notably, the median prostate volume in the PCa group was close to the suggested lower threshold of 39 mL under which a good clinical outcome is unlikely [25]. Accordingly, no volume reduction was observed and only one in three patients with PCa compared with every BPH patient demonstrated an immediate increase in PSA. However, five patients received hormone or anti-androgen therapy known to alter PSA synthesis which may limit the use as a marker of prostatic injury [26]. Anti-androgen treatment may also increase prostate cancer cells resilience to apoptosis as a response the acute hypoxia, but whether this explains the difference between the groups is unknown [27]. More evidence is needed, but these findings suggest that active treatment for advanced prostate cancer should be carefully considered when evaluating patients for palliative PAE.

Perioperative data were also registered, and an ad hoc analysis showed a strong relationship between the amount of embolic material administered and the increase in PSA following PAE both groups. Other studies have shown a greater effect using a refined embolization technique where an additional 30–50% embolic particles can be injected [20,28]. These data suggest that the interventional radiologists should emphasize on carefully injecting as much embolic material as possible while avoiding reflux and non-target embolization.

Limitations of this study include the small sample size, short follow-up and participant heterogeneity. Additionally, a surrogate measure was used to evaluate the treatment response. Even though several groups supports elevation in PSA following embolization as a predictor of clinical outcome, it cannot replace actual clinical improvements which would require longer follow-up to assess. Further, these findings represent the first experience at our institution with prostate CTP. A refinement of the scanning protocol may improve discrimination between the prostate and adjacent soft tissue increasing the precision of perfusion measurements. Finally, postprocessing perfusion measurements including the manual outlining of the prostatic contour are subject to inter—and intra-rater variability which was not evaluated in this study.

## 5. Conclusions

Prostate volume strongly correlated with treatment response and the response was greater with incremental amounts of injected embolic material. PBF was not associated with elevation in PSA and did not add information that could guide patient selection.

## Figures and Tables

**Table 1:** Baseline and follow-up measures.

	BPH (*n* = 7)				PCa (*n* = 9)				Overall, (*n* = 16)			
Variable	Median	Min.	Max.	*p*-Value	Median	Min.	Max.	*p*-Value	Median	Min.	Max.	*p*-Value
Age, years	77.1	64.1	88.7	–	70.7	65.5	85.7	–	72.5	64.1	88.7	–
PBF, mL/min/100 mL	61.2	32.3	94.3	–	77.9	62.0	138.5	–	73.5	32.3	138.5	–
1 month	60.3	38.3	97.6	0.73	67.7	45.4	106.2	0.20	64.2	38.3	106.2	0.21
PSA, ug/L	8.7	2.0	30.0	–	13.0	0.2	65.0	–	9.0	0.2	65.0	–
24 h	147.0	3.7	323.0	0.02	9.9	0.2	659.0	0.44	67.0	0.2	659.0	0.01
1 month	6.2	1.9	12.0	0.47	4.4	0.2	66.0	0.53	5.3	0.2	66.0	0.89
Prostate volume, mL	174.6	30.9	246.0	–	43.6	11.3	135.4	–	70.2	11.3	246.0	–
1 month	149.0	24.8	237.6	0.02	43.6	10.2	98.5	0.73	55.3	10.2	237.6	0.04

BPH, benign prostatic hyperplasia. PBF, prostatic blood flow. PCa, prostate cancer. PSA, prostate-specific antigen. -, not applicable.

**Table 2:** Correlation coefficients between 24-h change in PSA and variables.

Table 2	24-h Change in PSA					
	BPH		PCa		Overall	
Variable	*p*	*p*-value	*Ρ*	*p*-value	*p*	*p*-value
Baseline PBF	−0.25	0.59	0.22	0.58	−0.18	0.51
Baseline prostate volume	0.68	0.09	0.87	<0.01	0.87	<0.001
Prostate volume reduction	−0.18	0.70	−0.63	0.07	−0.72	<0.01
Embolic particles administered	0.88	0.01	0.70	0.04	0.53	0.04

BPH, benign prostatic hyperplasia. PBF, prostatic blood flow. PCa, prostate cancer. PSA, prostate-specific antigen. ρ, Spearman’s rank correlation coefficient.
